# The Role of Alpha-Synuclein Autoantibodies in the Induction of Brain Inflammation and Neurodegeneration in Aged Humans

**DOI:** 10.3389/fnagi.2022.902191

**Published:** 2022-05-26

**Authors:** Manoj Kumar Pandey

**Affiliations:** ^1^Division of Human Genetics, Cincinnati Children’s Hospital Medical Center, Cincinnati, OH, United States; ^2^Department of Pediatrics, College of Medicine, University of Cincinnati, Cincinnati, OH, United States

**Keywords:** aging, neuro-immune crosstalk, memory deficits, autoimmunity, alpha-synuclein

## Introduction

Aging is a major risk factor for developing neuroinflammation. As it progresses, neuroinflammation can cause neuron death in the brain, particularly in the hippocampus (Miyashita, 2004; Stephenson et al., 2018). This brain region is crucial for learning and memory function. Hence, aged humans who experience loss of neurons in this region exhibit frequent tendency of memory loss (Landfield et al., 1996; Bach et al., 1999; Miller and O’Callaghan, 2003; Eichenbaum, 2004; Panegyres, 2004; Gallagher et al., 2010). Aging and its association for the development of numerous brain diseases are continuously increasing in prevalence (Wyss-Coray, 2016). Increased plasma, cerebrospinal fluid (CSF), and brain level of alpha-synuclein (α-syn) and their association to microglial cells activation, pro-inflammatory cytokines production, neurodegeneration, and cognitive deficits have been observed in aged humans (Li et al., 2004; Chu and Kordower, 2007; Fjorback et al., 2007; Goldman et al., 2018; Stephenson et al., 2018). However, the exact mechanism by which such α-syn abnormalities trigger neuroinflammation in aged humans are poorly defined. Studies have shown that irregular accumulations and distributions of α-syn and/or the development of α-syn- reactive immunoglobulin G (IgG) autoantibodies are linked to the brain production of pro-inflammatory cytokines, i.e., interleukin1 beta (IL1β), IL6, and tumor necrosis factor alfa (TNFα), which lead to neuron death and memory deficits in several age-related neurodegenerative diseases, i.e., Alzheimer’s (AD), Parkinson’s (PD), multiple system atrophy (MSA), rapid eye movement sleep behavior disorder (RBD), frontotemporal lobar dementia (PLD), and dementia with Lewy bodies (LBD) (Spillantini et al., 1997; Baba et al., 1998; Zhang et al., 2005; Croisier and Graeber, 2006; Loeffler et al., 2006; Papachroni et al., 2007; van Rooijen et al., 2009; Mazzulli et al., 2011; Smith et al., 2012; Sardi et al., 2013; Kim et al., 2014; Allen Reish and Standaert, 2015; Pandey et al., 2016; Akhtar et al., 2018; Scott et al., 2018; Gregersen et al., 2021). Therefore, this current study explored the involvement of α-syn, α-syn-reactive IgG autoantibodies, and Fc gamma receptors (FcγRs) function in aging human nervous system.

## α-Syn in Aged Humans

Alpha-synuclein (α-syn) is a protein composed of a 140-amino acids with a total molecular weight of approximately 14 kDa, and is encoded by α-syn gene (*SNCA*) (Maroteaux et al., 1988; Spillantini et al., 1995). α-syn expression has been observed in different regions of the brain (e.g., hippocampus, presynaptic terminals of neocortex, and substantia nigra), in several nerve cells, (e.g., neurons, astrocytes, and oligodendrocytes), CSF, serum, plasma, and hematopoietic cells (Borghi et al., 2000; Richter-Landsberg et al., 2000; Mori et al., 2002; El-Agnaf et al., 2003; Kim et al., 2004; Miller et al., 2004; Barbour et al., 2008; Scherzer et al., 2008). Neuronal α-syn has been linked to the regulation of dopamine biosynthesis (Perez et al., 2002), synaptic vesicle mobility and recycling (Murphy et al., 2000; Burré et al., 2010; Scott and Roy, 2012), neurotransmitter release (Nemani et al., 2010), lipid metabolism (Jenco et al., 1998; Jo et al., 2000; Payton et al., 2004; Castagnet et al., 2005; Golovko et al., 2005; Narayanan et al., 2005; Barceló-Coblijn et al., 2007), and inflammatory responses (Sharon et al., 2003). The abnormal production and aggregation of α-syn trigger microglial cell activation and the massive generation of several pro-inflammatory cytokines (e.g., IL-1β, TNF-α, and IL-6), causing neurodegeneration and cognitive deficits in aged humans (Li et al., 2004; Chu and Kordower, 2007; Fjorback et al., 2007; Goldman et al., 2018; Stephenson et al., 2018). The identification of the mechanism by which such α-syn abnormalities develop brain inflammation in aged humans could also be helpful for better understanding of the disease mechanisms and development of anti-neuroinflammatory treatments for both healthy individuals and patients with brain diseases.

## α-Syn-Specific IgG Autoantibodies in Aged Humans

Immunoglobulin G (IgG), with a molecular weight of ∼150 kDa, is an antibody produced by plasma B cells in response to antigens (Bandilla et al., 1969). The serum concentration of IgG ranges from 7 to 18 mg/ml in healthy adults (Gonzalez-Quintela et al., 2008). The IgG antibody is composed of four polypeptide chains: two identical gamma (γ) heavy (H) chains of 50 kDa and two identical kappa (κ) or lambda (λ) light (L) chains of 25 kDa, connected together by the inter-chain disulfide bonds (Cobb, 2020). IgG- H chain comprises N-terminal variable (VH) and three constant domains (CH1, CH2, CH3), with the supplementary hinge region between the CH1 and CH2 domains (Cobb, 2020). Likewise, each of the IgG- L chain consists of an N-terminal variable domain (VL) and a constant domain (CL). The assembly of the IgG-L chain variable (VL) and constant (CL1) domains to the conforming IgG-H chain variable (VH) and constant (CH1) domains results the formation of the fragment antigen binding arm (Fab), which is responsible for the binding of antigens (Kabat et al., 1978; Potter, 1983). Two H chain – L chain heterodimers (HL) combine into a single antibody molecule through disulfide bonds (S = S) in the hinge region and non-covalent interactions between the CH3 domains. The portion of the antibody formed by the lower hinge region and the CH2/CH3 domains is called fragment crystalline (Fc) (Vidarsson et al., 2014). The Fab region of the IgG binds to specific antigen, whereas Fc region of the IgG binds to IgG receptor termed as Fc gamma R (FcγR) which is expressed on the surface of various immune cells (Karsten et al., 2012; Pandey, 2013; Pandey et al., 2017; Ben Mkaddem et al., 2019). Humans have four subclasses of IgG, (e.g., IgG1, IgG2, IgG3, and IgG4), all of which plays a critical role in biology of the health and disease (Schur, 1988; Pandey et al., 2003; Pandey and Agrawal, 2004; Iankov et al., 2006; Pandey, 2013; Vidarsson et al., 2014). Elevated levels of IgG autoantibodies to several brain elements (e.g., amyloid beta, myelin, optic nerve antigen, glutamic acid decarboxylase) have been observed in serum and/or CSF of patients with AD, multiple sclerosis (MS), cerebellar ataxias, Batten, autoimmune glaucoma, Stiff-person syndrome, and major depressive disorders (Polman and Killestein, 2006; Seehafer et al., 2011; Joachim et al., 2013; Maftei et al., 2013; Mitoma et al., 2017). Similarly, α-syn- specific IgG auto antibodies have been observed to have a role in the induction of neuroinflammation in PD, MSA, and RBD (Loeffler et al., 2006; Papachroni et al., 2007; Smith et al., 2012; Akhtar et al., 2018; Scott et al., 2018; Gregersen et al., 2021). Studies have also found significance in the levels of α-syn reactive IgG auto antibodies in aged patients’ serum and CSF (Smith et al., 2012; Heinzel et al., 2014; Brudek et al., 2017; Horvath et al., 2017; Akhtar et al., 2018; Folke et al., 2019). Additionally, age dependent increases of α-syn reactive IgG auto antibodies have been observed in humans (Koehler et al., 2013). Based on these findings, it is suggested that the abnormal production or aggregation of α-syn may be a trigger for production of IgG antibodies to α-syn. The ligation of such α-syn specific IgG antibodies to α-syn causes the formation of α-syn specific IgG immune complexes (α – syn – IgG ICs) in aged humans.

## FcγRs in Aged Humans

The FcγRs are the membrane molecules belonging to the immune tyrosine activation motif (ITAM)-associated receptor family, which recognize the Fc region of the different sub classes of humans IgG (IgG1, IgG2, IgG3, and IgG4) (Pandey, 2013; Ben Mkaddem et al., 2019). These subclasses of IgG have variable affinities for binding to the corresponding FcγRs (Pandey, 2013). Based on function, human FcγRs are divided into two classes, namely, activating FcγRs (e.g., FcγRI, FcγRIIa, FcγRIIc, and FcγRIIIa) and inhibitory FcγR, i.e., FcγRIIb or FcγRIIIb; GPI linked decoy (Ravetch and Bolland, 2001; Pandey, 2013). Activating FcγRs (apart from human FcγRIIa) signal through the specific Fcγ chain that contains ITAM. The ligation of IgG-ICs, by activating FcγRs, causes the tyrosine phosphorylation of ITAM to the cytoplasmic chain by the SRC kinase family (Src, Fyn, Fgr, Hck, and Lyn) (Ghazizadeh et al., 1994; Wang et al., 1994; Ravetch and Bolland, 2001; Pandey, 2013). Resultant phosphorylated ITAM bind to the spleen tyrosine kinase (STK), causing the STK phosphorylation. The phosphorylated STK causes a downstream activation of the linker for activation of T cells (LAT), multimolecular adaptor complexes (MAC), and the phosphoinositide 3-kinase (PI3K). Such activation of LAT- MAC - PI3K pathway leads to the development of membrane-docking sites for Bruton’s tyrosine kinase (BTK), along with the recruitment of phosphoinositide base (PI) and phospholipase C-gamma (PLC-γ) (Pandey, 2013; Pandey et al., 2017). The PLC-γ is the multi-domain phosphodiesterase which catalyzes the conversion of the phosphatidylinositol (3,4,5)-trisphosphate [PI(3,4,5)P_3_] to three byproducts, i.e., phosphoinositol 4,5 diphosphate [PI(4,5)_2_], Inositol 1,4,5 triphosphate (IP3), and diacyl glycerol (DAG), which galvanizes the calcium ion (Ca^++^) mobilization and cause the activation of rat sarcoma virus (RAS) – rapidly accelerated fibrosarcoma (RAF) - mitogen-activated protein kinase (MAPK) pathway. This then leads to cellular activation, pro-inflammatory cytokines production, and the tissue damage that may entail degenerative diseases (Rafiq et al., 2002; Nakashima et al., 2004; Matsubara et al., 2006; Nimmerjahn and Ravetch, 2008; Karsten et al., 2012). In contrast, inhibitory FcγRIIb crosslinking with IgG-ICs triggers the activation of immunoreceptor tyrosine inhibitory motif (ITIM)- downstream signaling and causes the tyrosine phosphorylation of ITIM by Lyn kinase, leading to the conversion of SH2 containing phosphatidylinositol 5 phosphatase (SHIP) to phosphorylated SHIP. Furthermore, the reaction between the phosphorylated SHIP and PI (3, 4, 5) P3 causes the suppression of PI (3, 4, 5) P3- mediated induction of cellular activation (Nimmerjahn and Ravetch, 2008; Pandey, 2013). Overall, the IgG-ICs ligation with activating FcγR causes the transduction of inflammatory signaling through ITAM, causing cellular activation, pro-inflammatory cytokines production, and cell death, which are all inhibited by the IgG-ICs- inhibitory FcγRIIb-ITIM-downstream signaling in humans (Nimmerjahn and Ravetch, 2007, 2008; Wang, 2019). These observations suggested that the proportion of activating vs. inhibitory FcγRs (A/I ratio) determines the magnitude of IgG-ICs-induced inflammatory cell responses (Ravetch and Lanier, 2000; Shushakova et al., 2002; Godau et al., 2004; Ricklin et al., 2012). The steady state human microglial cells showed lower expression of ITAM-FcγRs (e.g., FcγRI, FcγRIIa, FcγRIIb, and FcγRIIIa). In addition, compared to inhibitory FcγR, increased expression of activating FcγRs have been observed in choroid-retinal epithelial cells, hippocampal tissues, microglial cells, and neurons of healthy individuals and elderly patients with other neurodegenerative diseases (Peress et al., 1993; Ulvestad et al., 1994b; Mohamed et al., 2002; Andoh and Kuraishi, 2004; Orr et al., 2005; Kam et al., 2013; Zotova et al., 2013; Murinello et al., 2014; Yue et al., 2019). Additionally, age-associated increased production of α-syn reactive IgG auto antibodies and higher microglial cells expression of activating FcγR have been observed in the CNS regions of the healthy humans (Hart et al., 2012; Koehler et al., 2013). Based on these findings, it is speculated that the robust crosslinking between α-syn – IgG-ICs and the corresponding activating FcγR causes the activation of ITAM and its downstream signaling cascades, which trigger the microglial cells activation and massive generation of pro-inflammatory cytokines that lead to neuron death and cognitive defects.

## Discussion

Aging and its association with developing several of the age-related brain diseases such as AD, PD, MSA, RBD, Huntington (HD), amyotrophic lateral sclerosis (ALS), and PLD are continuously increasing (Budni et al., 2015; Wyss-Coray, 2016). Despite putting considerable effort, the mechanism that underlie age-related development of neuroinflammation, neurodegeneration, and cognitive decline has been poorly defined.

Mice IgG (IgG1, IgG2a/c, IgG2b, and IgG3) and their matching receptors, (e.g., FcγRI, FcγRIIb, FcγRIII and FcγRIV) are different from human IgG, (e.g., IgG1, IgG2, IgG3, and IgG 4) and receptors, (e.g., FcγRI, FcγRIIa, FcγRIIc, FcγRIIIa, FcγRIIb, and FcγRIIIb) (Pandey, 2013). Human or mice FcγRI bind only to the monomeric IgG, but all the other FcγR in mice, (e.g., FcγRIIb, FcγRIII and FcγRIV) and human, (e.g., FcγRIIa,FcγRIIb, FcγRIIIa and FcγRIIIb) can bind to IgG-ICs (Nimmerjahn and Ravetch, 2006). Mice IgG2a/c-ICs, when cross linked to FcγRIII/FcγRIV, causes immune inflammation (Clynes et al., 1999; Yuasa et al., 1999; Karsten et al., 2012; Pandey, 2013). However, mice IgG1-ICs, when crosslinked to FcγRIIb, develops protection against inflammation (Schur, 1988; Karsten et al., 2012; Pandey, 2013; Vidarsson et al., 2014). In contrast, human activating FcγR interaction with IgG1-ICs triggers immune inflammation, but their inhibitory FcγR ligation to IgG4-ICs protects against inflammation (Pandey, 2013). Moreover, the effector function of human IgG1 is like murine IgG2a/c. Similarly, the effector function of human IgG4 is more alike to mouse IgG1 (Pandey, 2013). Mice studies have shown the higher expression of FcγRIV and FcγRIIb receptors in females (Gal-Oz et al., 2019). However, human studies have revealed almost similar level expression of FcγRIIIa in male and females (Huang et al., 2021).

Despite having several of the indicated differences in IgG and their corresponding FcγRs in mice and humans, both mice and human studies have shown that the IgG-ICs cross linking to activating FcγR causes tissue inflammation (Clynes et al., 1999; Yuasa et al., 1999; Karsten et al., 2012; Pandey, 2013) and that IgG-ICs interaction to inhibitory FcγR protects IgG-ICs-activating FcγR axis-induced tissue inflammation (Nimmerjahn and Ravetch, 2007; Karsten et al., 2012; Pandey, 2013). Mice and human studies have also shown that the co-expression of FcyRs activation and inhibition are essential for establishment of the threshold that controls the activation of IgG-ICs – FcγR-mediated effector functions and the disturbances of such balance between the activating and inhibitory FcγR as well as their crosslinking to the corresponding IgG/IgG subclasses-ICs mechanistically provoke the immune inflammations and cause tissue damage in several inflammatory diseases (Nimmerjahn and Ravetch, 2007; Karsten et al., 2012; Pandey, 2013; Pandey et al., 2017).

Brain reactive antigens, autoantibodies, and their link to FcγR- mediated microglial cells activation, pro-inflammatory cytokines, (e.g., IL-1β, IL6, IL18, and TNFα) production, neurons death, and the memory and learning defects have been observed in several neurological diseases (Polinsky et al., 1991; Ulvestad et al., 1994a,b; Terryberry et al., 1998; Gruden et al., 2007; Vyshkina and Kalman, 2008; Okun et al., 2010; Lunnon et al., 2011; Cribbs et al., 2012; Satoh et al., 2012; Vacirca et al., 2012; Joachim et al., 2013; Kam et al., 2013; Murinello et al., 2014; Paris et al., 2014; Schweig et al., 2017, 2019; Yang et al., 2019). The number of the growth and neurotrophic factors, i.e., brain-derived neurotrophic factor (BDNF), nerve growth factor (NGF), and the glial cell line-derived neurotrophic factor (GDNF) are essential for pruning, myelination, differentiation, synaptic and neuronal growth, survival of neurons, and the scalp skin homeostasis (Huang and Reichardt, 2001; Skoff et al., 2003; Adly et al., 2006, 2016; Caldeira et al., 2007; Skaper, 2012; Mitra et al., 2021). Studies have shown the link between the abnormal brain formation of BDNF, NGF, and GDNF and the neurodegeneration that comes with age-related brain diseases (Grassi-Oliveira et al., 2008; Komulainen et al., 2008; Numakawa et al., 2018; Lima Giacobbo et al., 2019). However, the exact mechanism by which α-syn- induced initiation of IgG ICs - activating FcγRs axis triggers neuronal cells activation, pro-inflammatory cytokines production, and neurodegeneration was not explored in human nervous system aging. To resolve all these critical topics, direct *in vivo* and *ex vivo* functional studies demonstrating the α-syn-IgG-ICs – activating and/or inhibitory FcγRs axis- downstream signaling and their impact in controlling microglial cells and/or neurons activation, IL-1β, IL6, IL18, TNFα, BDNF, NGF, and GDNF production as well as their combined influence on the status of neurons health and memory function are needed for mice and/or humans with different ages and genders.

**FIGURE 1:**
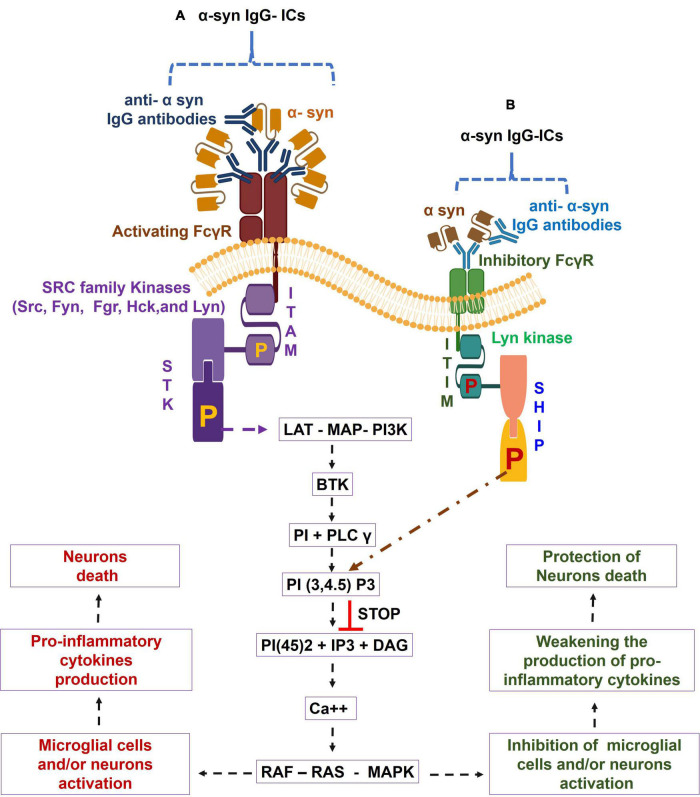
Alpha-synuclein-specific IgG immune complexes (α-syn-IgG-ICs) – Fc gamma receptor (FcγR) function in induction of neuroinflammation in aged humans. During aging, α-syn- IgG-ICs interaction with the activating FcγR activates the SRC family kinases, (e.g., Src, Fyn, Fgr, Hck, and Lyn). These enzymes trigger the phosphorylation of immune-tyrosine activation motif (ITAM) and cause the recruitment of SH2 domain having Syk kinases, which lead to the phosphorylation of spleen tyrosine kinase (STK). Such alteration in STK causes the activation of the linker for activation of T cells (LAT)- multimolecular adaptor complexes (MAC)- phosphoinositide 3-kinase (PI3K) – Bruton’s tyrosine kinase (BTK) – phosphoinositide base (PI) + phospholipase C-gamma (PLC-γ) – phosphatidylinositol (3,4,5)-trisphosphate**:** PI(3,4,5)P_3_ – phosphoinositol 4,5 diphosphate [PI(4,5)_2_] + Inositol 1,4,5 triphosphate (IP3) + diacyl glycerol (DAG) – calcium (Ca^++^) – rat sarcoma virus (RAS) – rapidly accelerated fibrosarcoma (RAF) – mitogen-activated protein kinase (MAPK) pathway and cause increased activation of microglial cells and/or neurons and pro-inflammatory cytokines generation, which lead to neuron death in aged humans **(A)**. Furthermore, due to aging-induced down regulation of inhibitory FcγR, α syn-IgG-ICs fail to establish proper binding to the inhibitory FcγR and, in turn, the inhibitory FcγR- downstream LYN kinases- phosphorylated immunoreceptor tyrosine inhibitory motif (ITIM) – phosphorylated SH2-containing-phosphatidylinositol-5′-phosphatase (SHIP) anti-inflammatory pathway do not control the α syn- IgG-ICs – activated FcγRs axis- mediated microglial cells and/or neurons activation, pro-inflammatory cytokines generation, and the neurons death in aged humans **(B)**.

However, based on the elevated brain expression of α-syn- reactive IgG auto antibodies and the higher expression of activating FcγR in aged humans (Hart et al., 2012; Koehler et al., 2013; Heinzel et al., 2014; Brudek et al., 2017; Horvath et al., 2017; Akhtar et al., 2018; Folke et al., 2019), this report suggests that the α- syn- reactive IgG auto antibodies and their immunological reaction to α-syn are the basis of the formation of the α-syn – IgG-ICs in the aged human brains. Furthermore, the strong interaction between such α syn- IgG-ICs and activation of FcγR fules the activating FcγR- downstream signaling that causes the microglial cells and/or neurons activation, pro-inflammatory cytokines production, and the neurons death in aged humans (Figure 1A). Also, downregulation of inhibitory FcγR in aging permits poor communication between α syn IgG – ICs and the corresponding inhibitory FcγR and their downstream ITIM and SHIP signaling that principally controls the α-syn – IgG-ICs – activating FcγR axis- downstream signaling-mediated microglial cells and/or neurons activation, pro-inflammatory cytokines generation, and the neurons death in aged humans (Figure 1B). Targeting the α syn- IgG-ICs – activating FcγR axis and/or their downstream signaling by which microglial and/or neuronal cells activate and fuel brain inflammation and develop memory and learning defects could help to understand the disease mechanisms and development of the alternative anti-neuroinflammatory treatments for aged humans.

## Author Contributions

MP has intellectualized and wrote the study, conducted the literature search, performed the proofreading, editing, and approval of final version of the manuscript.

## Conflict of Interest

The author declares that the research was conducted in the absence of any commercial or financial relationships that could be construed as a potential conflict of interest.

## Publisher’s Note

All claims expressed in this article are solely those of the authors and do not necessarily represent those of their affiliated organizations, or those of the publisher, the editors and the reviewers. Any product that may be evaluated in this article, or claim that may be made by its manufacturer, is not guaranteed or endorsed by the publisher.
